# Measurement of Cadmium Ion in the Presence of Metal-Binding Biopolymers in Aqueous Sample

**DOI:** 10.1155/2013/270478

**Published:** 2013-09-08

**Authors:** Jian Pu, Kensuke Fukushi

**Affiliations:** Integrated Research System for Sustainability Science (IR3S), The University of Tokyo, 7-3-1 Hongo, Bunkyo-ku, Tokyo 113-8654, Japan

## Abstract

In aqueous environment, water-soluble polymers are effectively used to separate free metal ions from metal-polymer complexes. The feasibilities of four different analytical techniques, cadmium ion-selective electrode, dialysis sack, chelate disk cartridge, and ultrafiltration, in distinguishing biopolymer-bound and nonbound cadmium in aqueous samples were investigated. And two different biopolymers were used, including bovine serum albumin (BSA) and biopolymer solution extracted from cultivated activated sludge (ASBP). The ISE method requires relatively large amount of sample and contaminates sample during the pretreatment. After the long reaction time of dialysis, the equilibrium of cadmium in the dialysis sack would be shifted. Due to the sample nature, chelate disk cartridge could not filter within recommended time, which makes it unavailable for biopolymer use. Ultrafiltration method would not experience the difficulties mentioned above. Ultrafiltration method measuring both weakly and strongly bound cadmium was included in nominally biopolymer-cadmium complex. It had significant correlation with the Ion-selective electrode (ISE) method (*R* ^2^ = 0.989 for BSA, 0.985 for ASBP).

## 1. Introduction

Water-soluble polymers play an important role in various industries producing superconducting materials, ultra-high strength materials, liquid crystals, catalysts, and biocompatible polymers, because of the metal ion interactions in homogeneous medium of environmentally benign solvent water [1, 2]. A number of studies pertaining to water-soluble biopolymers consisting of proteins, lipids, polysaccharides, nucleic acids, lipoproteins, glycocalyxes, have been reported to remove heavy metals from solutions as well as soils [3–9]. Metal, ligand (i.e., biopolymer), and metal-ligand complexes form a homogeneous system; therefore, some separation process is needed to distinguish between free metal ions and metal-polymer complexes [10].

In order to measure nonbound metal in presence of the metal bound to water-soluble polymers, a number of techniques have been applied for aqueous samples. Ion-selective electrode (ISE) is a typical and easy tool to measure the concentration of free metal ions in liquid phase [10]. Ions that can be measured by ISE include fluoride, bromide, and cadmium. Ion-exchange resins have been utilized to separate the aqueous samples into free metal ions, labile complexes, slowly-labile complexes, and stable complexes. The charged metal species exchange with the ions on the resins. Thereby it is possible to divide the sample into different species. Cation-exchange resins can be used to divide metal complexes by their stability, whereas organic and inorganic complexes can be separated by the use of anion-exchange resins [11]. Membrane filtration easily allows the separation of metal ions bound to soluble polymers from nonbound metals [1]. Dialysis sacks or tubes were applied for the separation of metal ions from mixture samples [11]. Liu et al. (2001) and Zheng et al. (2008) used dialysis membrane to retain metal-bound biopolymers from the aqueous sample for the measurement of biopolymer-bound metal [9, 14]. Ultrafiltration appears to be a versatile separation technique, which allows the separation of higher molecular weight (MW) polymers in presence of lower MW species, such as metal ions. Metal-bound polymers are not able to pass through the ultrafiltration membrane, while free ions are eluted through the membrane [2, 12, 15]. 

The authors of this paper are working on a project to utilize water soluble biopolymers extracted from waste activated sludge as an enhancement agent to separate heavy metal from contaminated soil. In order to evaluate the role of water soluble biopolymers on metal removal, precise measurement of metal-bound biopolymer is important. In this paper, several methods were tested to measure nonbound cadmium ions or bound cadmium in order to determine the concentrations of cadmium complex formed in the aqueous biopolymers.

## 2. Materials and Methods

### 2.1. Preparation of Water-Soluble Biopolymer

Water-soluble biopolymer was extracted from cultivated sludge grown in a nonselective medium. The returned sludge from municipal wastewater treatment was introduced to culture medium and the batch cultivation was done at 120 rpm in 25°C thermostat for 24 h. The composition of the medium was as follows (in mg/L): glucose, 4675; peptone, 1000; K_2_HPO_4_, 3000; KH_2_PO_4_, 1500; NH_4_Cl, 1125; MgSO_4_·7H_2_O, 562.5; FeSO_4_·7H_2_O, 18.75; ZnSO_4_·7H_2_O, 18.75; MnSO_4_·7H_2_O, 18.75; CaCl_2_, 75; NaHCO_3_, 900. The culture liquid was centrifuged (10,000 g, 5 min, 4°C) and the pellet was resuspended in 20 mM of Tris-HCl buffer (pH 8). Lysozyme was added, since as a lytic enzyme treatment, it is a viable alternative or complement to mechanical or chemical methods of cell disruption where the isolation of an intracellular component is best initiated by a gentle, highly specific procedure [16]. Then the suspension went through freeze and thaw for 3 cycles at −80°C and 30°C. Later, the suspension was sonicated for 15 min with 50% burst at 170 W, 20 kHz in ice-water bath to prevent the increase of the sample temperature. The supernatant was filtered through 0.45 *μ*m membrane. The permeate containing water soluble biopolymers was used for metal binding experiment. The protein concentration was about 2.8 mg/L measured by Lowry method [17], with bovine serum albumin (BSA, MW = 66 kDa) as standard. BSA was also used as a reference water-soluble biopolymer.

The MW of activated sludge biopolymer (ASBP) was analyzed by sodium dodecyl sulfate polyacrylamide gel electrophoresis (SDS-PAGE) with Coomassie brilliant blue (CBB) R250 staining. Raw data were purchased from Takara Shuzo Co., Ltd. (Shiga, Japan). A broad range of MW was found in ASBP, as summarized in Table 1. About 21.4% of ASBP had MW over 50 kDa (3.9% over 100 kDa) and 10.5% below 5 kDa. The mean MW of ASBP was 30 kDa, calculated from Table 1. The results of MW distribution obtained by this measurement were considerably lower than the 200 kDa reported for extracellular polymeric substances extracted from activated sludge by physical treatment followed by removal of low MW solutes lower than 1 kDa [14]. 

### 2.2. Cadmium Adsorption Experiment

Cadmium solutions were prepared by dissolving Cd(NO_3_)_2_ in water at 1000 mg/L to make the super stock solution. The super stock solution was diluted to a desired concentration range of 0.5–10 mg/L. A series of adsorption tests was conducted at neutral pH in 50 mL of polypropylene test tubes at ambient temperature (about 25°C). The cadmium binding reagents (EDTA, BSA, ASBP) were spiked to test tubes containing cadmium solution for a series of adsorption tests. The cadmium concentrations used were 0.5, 1, 2, 5, and 10 mg/L, which equaled to 4.2 × 10^−3^, 8.4 × 10^−3^, 1.7 × 10^−2^, 4.2 × 10^−2^, and 8.4 × 10^−2^ mM. The concentration of biopolymers was approximately 2 × 10^−3^ mM for both BSA and ASBP, while it was 62 mg/L (approximately 0.2 mM) for EDTA to ensure an excess of EDTA compared to cadmium added by assuming one mole of EDTA binds with one mole of cadmium. Cadmium uptake was expressed as the amount of cadmium bound with cadmium binding reagent over the control run which does not contain cadmium binding reagents. Test tubes were gently agitated on a reciprocal shaker at 40 rpm for 24 hours.

The ionic concentration in the mixture was measured by ion-selective electrode (ISE) for cadmium (Orion, Model 9648BNWP, USA). The mixed liquor in the presence of biopolymers was then analyzed for cadmium using ISE and three separation methods in combination with ISE. All the separation methods were described in the next section.

### 2.3. Measurement Methods of Biopolymer-Bound Cadmium

The separation measurement of biopolymer-bound cadmium was determined by ISE, dialysis membrane, chelate disk cartridge, and ultrafiltration.

#### 2.3.1. ISE

A solid-state cadmium ISE was used. The concentration of free cadmium ions was determined by the electrode potential from the calibration data developed by standard cadmium solution. The amount of cadmium bound to the biopolymers was determined by subtracting the amount of free metal from the amount of cadmium added.

#### 2.3.2. Dialysis

Three mL of liquid with biopolymer and cadmium was transferred to the dialysis sack (SnakeSkin dialysis tubing, 3.5 kDa cut-off, Pierce). The dialysis sack was sealed and placed into a 500 mL conical flask containing 350 mL of 100 mM PBS (phosphate buffered saline). After 2 h, the old dialysate was discarded and replaced with fresh one. Two hours later the dialysate was replaced with the same amount of fresh PBS and stayed overnight. The content of the residual cadmium in the sack was considered to be cadmium-biopolymer complex.

#### 2.3.3. Chelate Disk Cartridge

Chelate disk cartridge (Empore, iminodiacetate functionalized poly(styrene divinylbenzene)), a cation-exchange resin, was used to separate the much stable form of cadmium-biopolymer complexes. Five mL of 3.0 M nitric acid and 5 mL of Milli-Q water were sequentially passed through the cartridge. Then, 3 mL of the liquid with biopolymer and cadmium was passed through the cartridge, and 5 mL of Milli-Q water was passed through to rinse the cartridge. The 8 mL of leachate was collected and determined for cadmium with flame atomic absorption spectrometer (FAAS, Shimadzu AA-6200). The cadmium detected by FAAS is considered to be stable cadmium-biopolymer complex.

#### 2.3.4. Ultrafiltration

For ultrafiltration, the mixed liquor was introduced to 3 kDa cut-off using Amicon ultra-4 3 K device (Millipore) and washed with 1 mM PBS buffer for 3 times. Subsequent determination of the metal content in the filtrate was carried out by FAAS. The residual metal in the filtrate was then compared with original solutions; the difference represented the quantity of the metal adsorbed by the biopolymer that was retained by the membrane.

For all separation measurement approaches, the amount of cadmium-biopolymer complex forms in one liter of the aqueous solution was calculated and compared.

Chelate disk cartridge is packed with Chelex-100 resin, a chelating ion-exchange resin with functional iminodiacetic acid (IDA) groups in a styrene-divinylbenzene matrix. Chelex-100 (sodium form, 50–100 mesh, Sigma) was also tested as an adsorbent for metals in aqueous samples. The resin was initially treated with a 0.5 M sodium acetate buffer to control pH and alkalinity. Three mL of 10 mg/L cadmium solution and 0.5 mL of 2000 mg/L BSA were added to each test tube. The test tubes were agitated on a reciprocal shaker at 120 rpm. After 4 hours, 50 mg chelex-100 was added to the aqueous samples in each tube. And then the mixtures were gently agitated at 20 rpm for 5 min, 10 min, 30 min, 2 h, and 24 h before the separation of the chelex-100 and the supernatant. Subsequent determination of the cadmium content in the supernatant was carried out by FAAS.

All chemicals used, unless otherwise stated, were of analytical reagent grade. All chemical solutions were prepared with Milli-Q water. FAAS measurement was applied according to standard methods [18]. 

## 3. Results and Discussion

### 3.1. Cadmium-Binding Characteristics of Biopolymers

The ISE consists of a sensing element which is bonded into an epoxy body. When the sensing element comes in contact with the solution containing cadmium ions, an electric-potential is developed across the sensing element. This potential depends on the level of the activity or “effective concentration” of the free cadmium ions in solution.

**Figure 1:**
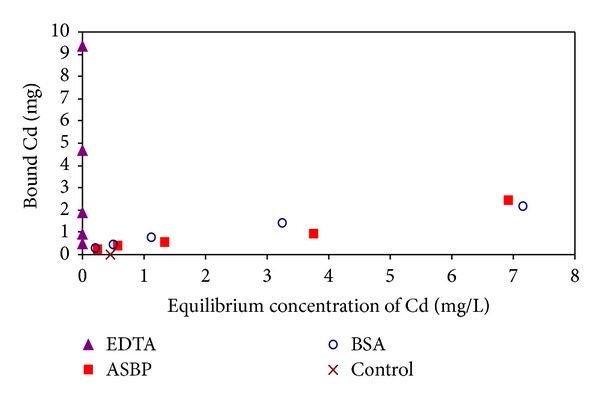
Different Cd-bound forms measured by ISE in 1 L solutions at different equilibrium concentrations. EDTA stands for strong chelating agent; BSA stands for protein with metal-binding efficiency; the bound cadmium in the later four concentrations in control series were under detection limit.

The EDTA- and biopolymer-bound cadmium complexes concentrations determined by ISE method were shown in Figure 1. At the start of isotherm experiment, the molar concentration of EDTA was set to be 2–40 times higher than cadmium. The results indicate that the EDTA complexed cadmium in the solution at any initial cadmium concentrations applied in this experiment. EDTA complex could not be detected by ISE. The concentration of biopolymer-bound cadmium increased with equilibrium concentration of cadmium. There are various types of functional groups, including ion exchange, chelate, and physical adsorption, in proteinaceous biopolymers. The results shown in Figure 1 indicated that biopolymers complex metals not only by chelete but also by adsorption. The isotherm experiment results, Figure 1, fitted well with Langmuir isotherm model (*R* ^2^ = 0.992 and 0.952 for BSA and ASBP, resp.). This indicates that a binding mechanism, follows to monolayer adsorption, was dominant especially at higher cadmium concentration for BSA and ASBP.

The concentrations of biopolymers (BSA and ASBP) bound with cadmium were determined between those of EDTA and the control; this indicates that cadmium was partially detected by ISE. It is suggested that biopolymers might be very tightly, weakly, and very weakly bound with cadmium, according to the strength and nature of cadmium-binding sites. According to the results obtained by the ISE method, the stability constants of the biopolymers, K(Cd-BSA) and K(Cd-ASBP), were calculated to be 2043 and 5164 mL/g, respectively, while the stability constant of EDTA-Cd complex known to be 2.9 × 10^16^ mL/g [19].

### 3.2. Measurement of Cadmium, Bound with Biopolymers Using Different Methods

**Figure 2:**
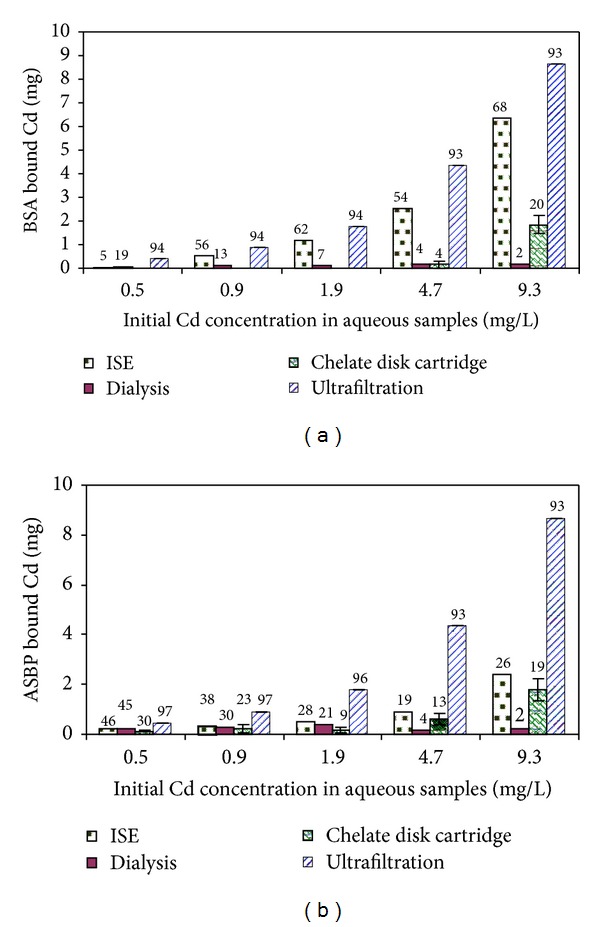
Comparison of biopolymer-bound cadmium using four different analytical techniques (ISE, dialysis, chelate disk cartridge and ultrafiltration), in 1 L solution (a) BSA; (b) ASBP. The values on the columns represent the percentage of biopolymer-bound cadmium to total added cadmium before adsorption test. Error bars represent observed low and high values for duplicate experiments.

The concentrations of cadmium complex formed in the aqueous biopolymers samples were shown in Figure 2, using the ISE method, dialysis sack, chelate resin disk cartridge, and ultrafiltration as separation measurement approaches.

Results of ISE were considered as reference value for other measurements. The disadvantage of ISE method is that it requires relatively large amount of samples; in addition, contamination of sample may occur during the pretreatment of sample using this method, since KNO_3_ is required to be added into samples as an ionic strength adjuster, at a volume ratio of KNO_3_ to sample as high as 1 : 10. In this work, ultrafiltration provided the highest concentration of cadmium-biopolymer complex measured at any initial cadmium concentration.

As discussed earlier, biopolymers have various functional groups on the surface which might bind tightly or weakly with cadmium, according to the strength and nature of cadmium-binding sites. In Figure 2, cadmium-biopolymer complex measured by ISE showed lower values compared to those by ultrafiltration-FAAS for BSA and ASBP. This may be due to the reason that weakly-bound cadmium by biopolymers would appear as nonspecific binding to be detected as the effective concentration in solution by ISE. In ultrafiltration method, the ratios of cadmium to biopolymer were calculated from Figure 2 to be 3–21 for BSA and 2–39 for ASBP, respectively, (data not shown here). The stoichiometric ratios in the literature showed 1–10 cadmium per biopolymer which corresponded to nonsurface-bound binding [5, 7, 20–22]. Ligand complexed or chelated metals are referred to as “specific binding” in the literature [23]. However, in addition to the specific bindings, proteins employ various functional groups that have potentially associated with metals. The literature report as high as 83–465 mole cadmium per mole biopolymer [24, 25]. The results of UF method shown in Figure 2 include both specific and nonspecific bindings. 

Interactions of metal ions with water-soluble polymers were mainly due to electrostatic forces and the formation of coordinating bonds. Other weak interactions might appear such as the trapping of metal ions in the bulk of the polymer phase [1]. During the long reaction time of dialysis (16 hours), the equilibrium concentration of cadmium in the dialysis sack would be gradually changed by dilution. The cadmium ion concentration in the dialysis sack decreased gradually, and weakly-bound cadmium on the surface of biopolymer released after the 16 hours dialysis. Moreover, the released cadmium was escaped through the dialysis membrane which encouraged the further release of cadmium from the biopolymer surface.

**Figure 3:**
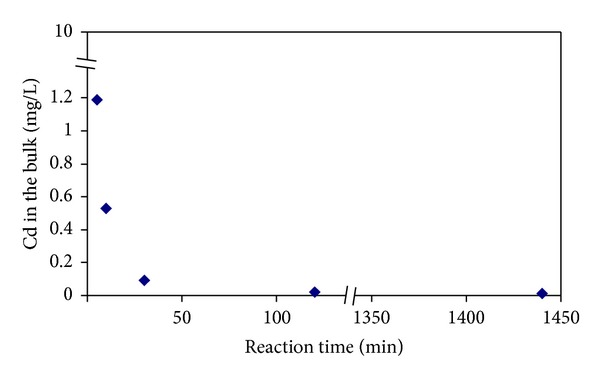
Time effect of Chelex-100 on cadmium species in BSA-cadmium solutions.

A fast flow with a flow rate of 10 mL/min is recommended for Chelate disk cartridge by the manufacture. However, samples with biopolymer could not pass through the disk within the recommended time, due to the sample nature. The flow rate varied from 0.3 to 6 mL/min at a vaccum pressure of 3.4 × 10^4^ Pa. In the other hand, chelate disk cartridge was found to be highly sensitive with filtration time, which brought a great fluctuation in cadmium concentration in the filtrate. In order to test the time influence in the chelate disk cartridge, the package material of chelate disk cartridge, Chelex-100 was added into BSA-cadmium solutions and cadmium in the bulk solution was measured by FAAS after different reaction times (Figure 3). The concentrations of free cadmium ions were 1.19, 0.53, 0.09, and 0.02 mg/L at the reaction times of 5, 10, 30, and 120 min, respectively. As shown in Figure 3, the concentrations of free cadmium ions were influenced largely by the reaction time, in addition it took long, various, and uncontrolled time for biopolymers to fulfill the filtration; therefore, the chelate disk cartridge is not recommended for the separation of heavy metals bound with biopolymers from the aqueous samples.

In order to investigate the rate limiting steps in the removal process of cadmium by chelex-100, kinetic models were studied through data fitting of the experimental data, including the equation of Lagergren pseudo-first-order kinetics, the pseudo-second-order kinetics, and the intraparticle diffusion model [13]. The pseudo-second-order had the best fitting, with *q*_*e*_ = 0.60 mg/g, *k*_2_ = 2.11 g/(mg·min), *R* ^2^ = 1.000, where *q*_*e*_ is the amount of adsorption by the biosorbent at equilibrium and *k*_2_ is a second-order speed constant of biosorption [26]. The results indicated that the adsorption was not diffusion controlled, but chemisorption (data not shown). The results indicated that the adsorption was not diffusion controlled but chemisorption (data not shown).

For both BSA and ASBP, ultrafiltration method had significant correlation with those of the ISE method (*R* ^2^ = 0.989 for BSA, 0.985 for ASBP, data not shown). No other significant correlations were found among ISE method, dialysis, and ultrafiltration with the condition applied in this experiment.

## 4. Conclusions

Water-soluble biopolymers extracted from a laboratory activated sludge showed binding capacity with cadmium under various initial cadmium concentrations. Four different methods were investigated to analyze biopolymer-bound cadmium, which is water soluble, using ion-selective electrode (ISE), dialysis, chelate disk cartridge, and ultrafiltration. The ISE method requires relatively large amount of samples and contaminates sample during the pretreatment. After the long reaction time of dialysis, the equilibrium of cadmium in the dialysis sack would be shifted. Due to the sample nature, chelate disk cartridge could not filter within recommended time, which makes it unavailable for biopolymer use. Ultrafiltration method would not experience the difficulties mentioned above, and it had significant correlation with the ISE method (*R* ^2^ = 0.989 for BSA, 0.985 for ASBP). Ultrafiltration method measured both weakly and strongly bound cadmium as biopolymer-cadmium complex. This method can be applied in distinguishing biopolymer-bound and nonbound cadmium in aqueous samples.

## Figures and Tables

**Table 1:** Molecular weight distribution of activated sludge biopolymer (ASBP). MW fraction was calculated based on the band intensity of each protein band of CBB-stained SDS-PAGE gel.

Molecular weight (kDa)	Fraction (%)	Molecular weight (kDa)	Fraction (%)
<3	10.0	30~50	12.4
3~5	0.5	50~60	8.4
5~10	8.6	60~80	3.7
10~20	38.4	80~100	5.4
20~30	8.6	>100	3.9
